# Spatial and temporal analysis of tuberculosis in Zhejiang Province, China, 2009-2012

**DOI:** 10.1186/s40249-016-0104-2

**Published:** 2016-02-23

**Authors:** Erjia Ge, Xin Zhang, Xiaomeng Wang, Xiaolin Wei

**Affiliations:** School of Biomedical Sciences, The Chinese University of Hong Kong, Hong Kong, S.A.R. China; The Chinese University of Hong Kong, Shenzhen Research Institute, Shenzhen, China; TB Program, Zhejiang Centre for Disease Control and Prevention, No. 630 Xincheng Road, Binjiang District, Hangzhou, Zhejiang 310051 China; JC School of Public Health, The Chinese University of Hong Kong, 2/F, School of Public Health, Prince of Wales Hospital, Shatin, Hong Kong, China; Dalla Lana School of Public Health, University of Toronto, 155 College Street, Toronto, ON M5T 3 M7 Canada

**Keywords:** Tuberculosis, spatial analysis, space-time clusters, Zhejiang, China

## Abstract

**Background:**

Tuberculosis (TB) is an infectious disease of major public health concern. The disease has demonstrated large space-time variations. This study aims to explore the space-time dynamics of TB cases in an economically and geographically dynamic province in China with specific references of TB control for policy makers.

**Methods:**

Data on all reported TB cases from 2009 to 2012 were collected from the TB program at the Zhejiang Provincial Center for Disease Control and Prevention. We employed time series and exploratory spatial data analyses, including Moran’s *I*, Local Getis’s *G*_i_^*^, and Kulldorff’s space-time scan statistics, to identify the temporal trends and spatial patterns of TB at a county level.

**Results:**

A total of 147,941 TB cases were reported during 2009–2012 in Zhejiang. A higher proportion of TB cases were younger, male, and registered permanent residents among all TB cases notified in the province. TB cases were reported most frequently in April with small peaks in June, July, and October. This disease was spatially clustering with Moran’s *I* values ranged from 0.29 to 0.32 (*p* < 0.001). A most likely cluster and ten secondary clusters were identified, mainly concentrated in the southeast and west counties of the province.

**Conclusions:**

This study identified seasonal patterns and significant space-time clusters of TB cases in Zhejiang, China. Poverty, migration, and seasonal effects may play important roles in potential clusters.

**Electronic supplementary material:**

The online version of this article (doi:10.1186/s40249-016-0104-2) contains supplementary material, which is available to authorized users.

## Multilingual abstracts

Please see Additional file 1 for translations of the abstract into the six official working languages of the United Nations.

## Background

Tuberculosis (TB) is an infectious disease caused by the bacteria called *mycobacterium tuberculosis.* Over 80 % of global new cases were reported in developing countries. China, with 855,000 TB cases or 14 % of world cases in 2013, has the second largest burden of TB cases in the world [1].

TB demonstrates highly complex dynamics and spatially heterogeneous in China at the national level [2–5] and the provincial level [6, 7]. Zhejiang is a province located in eastern China with relatively good economic development. The overall notification rate of TB in Zhejiang has remained as the middle level in China [8], but some regions (i.e., Quzhou and Wenzhou) in the province still suffer high risk of TB disease. Until now, few studies have investigated the space-time dynamics of TB clusters at the county level in China.

Many studies attempted to uncover the spatial patterns of TB cases under province, nationwide, or internationally during certain periods [3, 7]; however, they did not reveal small-area variations due to the use of relatively large scales. This study aims to investigate the space-time dynamics of TB clusters using a finer scale at a county level. We first examined the temporal trends and spatial patterns of TB cases between 2009 and 2012, using time series and advanced spatial statistical analyses. Space-time scan statistic analysis, commonly used for the detection of disease hotspots [9–11], was applied in the identification of TB clusters across different space and time. Geographical information systems (GIS) were used to map the analysis results.

## Methods

### Data collection

There are 54 million population in the province of Zhejiang, covering an area of 100,000 *km*^2^, including plain, mountains, seashore, islands and lakes [12]. We obtained the TB cases from the Zhejiang provincial TB program of the Zhejiang Provincial Center for Disease Control and Prevention (Zhejiang CDC). All these cases were reported to a nationwide infectious disease online reporting system and verified by the provincial and national TB program [13]. Each case contained information on age, gender, original residence, current address, date of TB symptom onset, date of diagnosis, and results of smear microscopy. Patients’ names and resident ID number were excluded because of privacy and confidential issues.

A number of 147, 941 confirmed TB cases were geocoded in terms of their residential addresses through matching each nominal address (completed with five to six hierarchical administrative district names) against the gazetter records and determining its longitude and latitude coordinates through the *Google* geocoding service and the toolbox of Geoprocessing in ArcGIS v.10.1 (ESRI Inc, Redlands, CA, USA). This process of geocoding was similar to previous studies applied in previous studies [14, 15]. We aggregated the TB cases by county in terms of locations of their homes.

### Statistics analysis

(a) Time Series and Descriptive Analysis

**Fig. 1:**
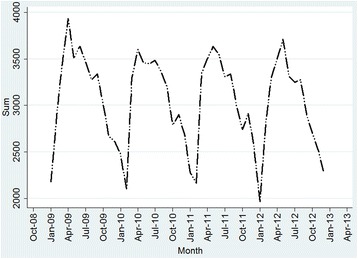
The monthly fluctuation of TB cases in Zhejiang province, China from 2009 to 2012

We aggregated all the TB cases by month in terms of their diagnosis dates to identify the temporal patterns of the disease. The time series included 48 months in total from January 2009 to December 2012 and was examined using *STATA 12* (Fig. 1). We also aggregated the TB cases regarding gender, age, and the year of infection to identify the demographic characteristics of the disease by years.

Time series and relevant descriptive analyses were conducted at the provincial level as all the TB cases reported in Zhejiang province during the period between 2009 and 2011 were included in our analyses. The advantage of conducting the time series analysis at the provincial level, compared with at the county level, is that this would provide a complete pattern for the disease dynamic in the province.

(b) Spatial Pattern Analysis

Global Moran’s *I* is a measure of spatial autocorrelation proposed by Moran [16]. This method has been widely used in spatial epidemiological studies [17, 18] The values of the Moran’s *I* range between -1 and 1, with 1 for maximum positive association and -1 for maximum negative association. There is no correlation when the value is zero. A higher positive value indicates a stronger spatial autocorrelation, and vice verso for negative values. We applied this method to identify the global pattern of the TB cases at the county level in Zhejiang province. The Z-score test was used to evaluate the significance of the estimate of the Moran’s *I*. A disease is regarded spatially aggregated and statistically clustered when the Moran’s I > 0 and Z-score ≥1.96 [19].

Local Getis’s *G*_i_^*^ statistic [20], a kind of local indicator of spatial autocorrelation [21] was also used in this study to identify the clusters (or hot sports) of TB cases. This statistic measures the degree of association that results from the concentration of disease cases within a certain distance from the location of the county. A significant positive value, e.g., *G*_i_^*^ ≥1.96, implies a clustering with a higher value locally than other areas, and a low clustering as the value *G*_i_^*^ ≤ - 1.96 [20]. In another word, a significant positive cluster could be identified through the estimate of *G*_i_^*^ statistic when several neighboring counties aggregate geographically with similar TB notification rates that were higher than those of other counties. We conducted 999 permutations to ensure the statistical significance at 95 % confidence intervals.

(c) Space-time Scan Statistic

Regarding the dimensions of space and time, we identified the clusters of TB across different counties geographically and periods of different time spans using SaTScan™ v7.0.2, a space-time scant statistic developed by Kulldorff [22]. This method defines a cylindrical window with a circular geographic base (i.e., a two dimension space) and with height corresponding to time. The cylindrical window moves over space and time scanning for clusters within the space-time window in order to compare the incident rates of TB outside this window. The null hypothesis was that the relative risk (*RR*) of TB cases within the window was equal to that of outside. We applied Poisson-based model to formulate the process of TB occurrence regarding a known underlying population at risk. The maximum size of space-time clusters should be decided when the most likely cluster identified is very large in size and contains smaller clusters that are statistically significant on their own strength [23], which may present more detailed and valuable information to uncover the aggregation of TB cases. In this study, the maximum size of space-time clusters was defined as twice higher risk at lest within the window than outside (i.e., *RR* ≥ 2) and there were two reasons for the selection. First, people within the identified clusters are more likely infected with TB bacteria than those of outside the clusters when the *RR* is greater than one. That is, this cluster is a risk factor for the infection of TB. On the contrary, the cluster is a protective factor when the *RR* is less than one. If no any effect (i.e., infection risk) on the infection, the *RR* is one. The selection for the maximum size of space-time cluster, therefore, needs the *RR* > 1 at least. Second, excessive relative risk (*ERR*) is an index measures how much the risk of, for example infection with TB, increases in association with a unit increase of the factor such as the size of the cluster. The relationship between *ERR* and *RR* is *ERR* = *RR*-1 and thus the value of the *RR* in this study should be equal or greater than two. We examined the significance of the identified cluster at a 95 % confidence level using Monte Carlo Simulation. The space-time scan window with the maximum likelihood value was the most likely cluster and other significant windows were called secondary clusters [24].

### Ethical review

In this study, TB data were collected by routine disease surveillance and control activities. We did not conduct any human subject research, and therefore the institutional review board approval was not required.

## Results

### Descriptive analysis of TB cases

A total of 147,941 TB cases were obtained in Zhejiang province from 2009 to 2012. The notification rates of TB in the province appeared to decrease from 89.26 in 2009 to 83.50 per 100,000 people in 2012, with the annual average rate of 84.11 per 100,000 people. In Table 1, the number of male cases was twice of that of female cases in any given year. In addition, a significant share of TB infections happened among adults between the age of 15 to 30 years old (over 30 %) and 30 to 45 years old (around 25 %). Among the reported cases, around two thirds were registered permanent residences while one third were migrants in Zhejiang.

**Table 1:** The demographic characteristics of TB cases in Zhejiang, China from 2009 to 2012

	2009	2010	2011	2012	Total
Gender					
Male	26674 (69.15)	25163 (68.23)	25095 (68.30)	24357 (68.14)	101289 (68.47)
Female	11899 (30.85)	11714 (31.77)	11649 (31.70)	11390 (31.86)	46652 (31.53)
Age					
0–15 year	357 (0.93)	340 (0.92)	316 (0.86)	300 (0.84)	1313 (0.89)
15–30 year	11650 (30.20)	11744 (31.85)	12192 (33.18)	11360 (31.78)	46946 (31.73)
30–45 year	9846 (25.53)	9296 (25.21)	8983 (24.45)	8354 (23.37)	36479 (24.66)
45–60 year	7816 (20.26)	7139 (19.36)	7395 (20.13)	7460 (20.87)	29810 (20.15)
>60 year	8904 (23.08)	8358 (22.66)	7858 (21.39)	8273 (23.14)	33393 (22.57)
Residence					
RPR	25732 (66.71)	24510 (66.46)	23877 (64.98)	23810 (66.61)	97929 (66.19)
RTR	12841 (33.29)	12367 (33.54)	12867 (35.02)	11937 (33.39)	50012 (33.81)

*RPR* Registered permanent residence, *RTR* Registered temporary residence

### Temporal patterns of TB cases

Figure 1 was daily time series of TB cases during 2009–2011. There was a strong seasonal pattern with large number of TB occurrence in April but decrease in winter. Mostly, the disease peaks were identified in summer between June and July, and late autumn.

### Spatial patterns of TB cases

**Fig. 2:**
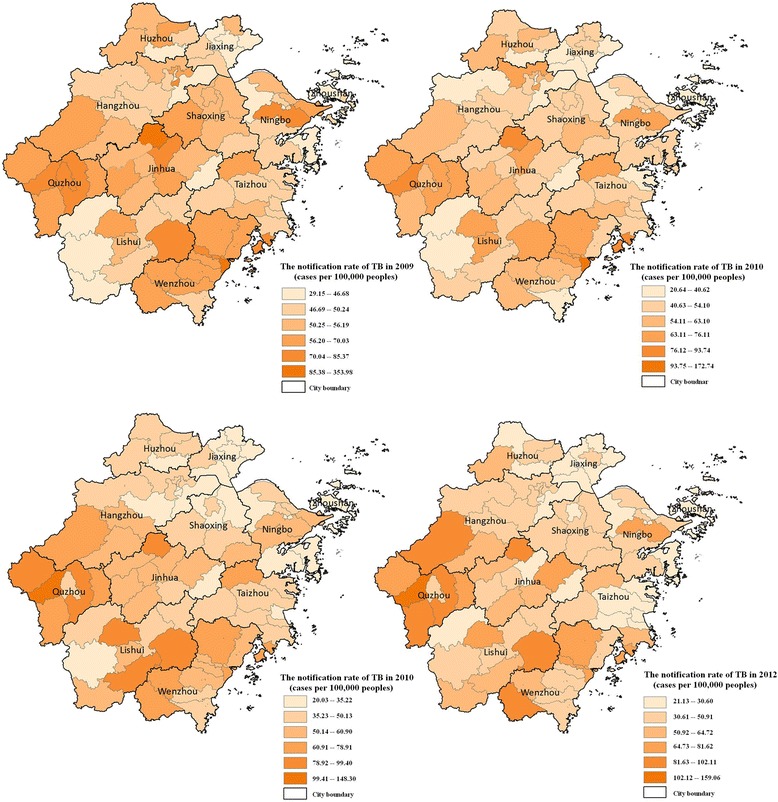
The notification rates of TB (the number of TB cases per 100,000 population) at a county level in Zhejiang province, 2009–2012

Figure 2 showed the spatial variations of TB notification rates between 2009 and 2012 at the county level in Zhejiang province. The cut-off values were determined through natural breaks (Jenks), which is a statistical method to ensure a minimum difference in values within a category but maximum differences between other categories. The notification rates of TB were significantly different in those years, and thus the cut-off values could not be the same. The highest notification rates were found in the counties of Quzhou and Wenzhou, respectively, in the west and southeast hilly parts of the province. The rates were relatively low in east and northeast counties, such as Jiaxing and Taizhou, where most areas are plains. The disease demonstrated highly dynamic and heterogeneous in space and time. The estimate of Moran’s *I*, for example, showed that TB were spatially auto-correlated across the period of the four years (Moran’s *I* ~ 0.3, *P* < 0.001, Table 2).

**Table 2:** Global spatial autocorrelation analyses for annual TB incidence rate in Zhejiang, China from 2009 to2012

Year	Moran’s I	Z-score	P value	Pattern
2009	0.296	6.356	<0.001	cluster
2010	0.315	8.268	<0.001	cluster
2011	0.307	5.829	<0.001	cluster
2012	0.323	8.137	<0.001	cluster

**Fig. 3:**
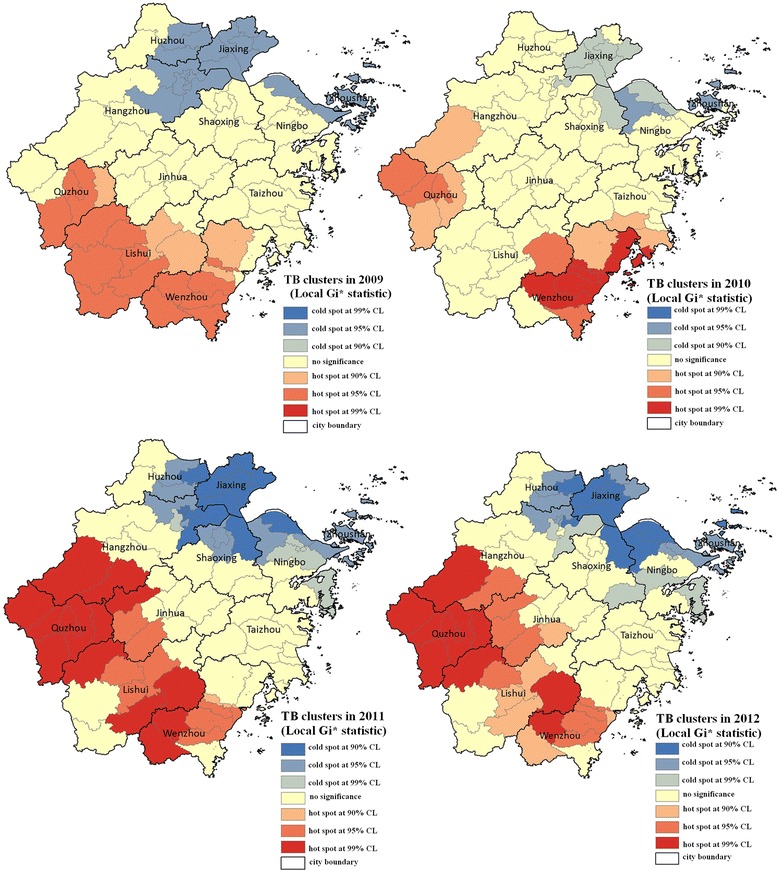
The spatial clusters (i.e., hot spots and cold spots) of the TB cases at the county level using the Local *G*_i_^*^ statistic

Figure 3 showed the analysis result of the Local Getis’s *G*_i_^*^ statistic. The clusters of TB cases, including hot spots and cold spot, were identified and mapped in terms of the estimates of the *G*_i_^*^ statistic and the False Discovery Rate (FDR) correction (*G*_i_^*^ z-score test) which defines a significant cluster at 90 %, 95 %, or 99 % confidence level in correspondence to the values of *G*_i_^*^ z-score test ±1.56, ±1.96, and ±2.58, respectively. The cluster is hot spot as the *G*_i_^*^ z-score ≥ +1.56, but cold spot when the *G*_i_^*^ z-score ≤ -1.56. There are no significance if the *G*_i_^*^ z-score is larger than -1.56 but less than +1.56. A “hot spot” occurs where the TB notification rates in a county as well as its neighboring counties are all high, relative to the distribution of the rates across all the counties of Zhejiang province. A county, for example, with *G*_i_^*^ z-score greater than 1.96 is a statistically significant “hot spot” at a 95 % confidence level. Conversely, a “cold spot” occurs where the notification rates in one county and its neighboring counties are all low, relative to the distribution of the incident rates across all the counties of the province. A county with *G*_i_^*^ z-score less than -1.96 is a statistically significant “cold spot” at a 95 % confidence level. The cut-off values were determined in terms of the *G*_i_^*^ z-score values, which were also applied across the four years in Fig. 3.

From our analysis result, we found that the most significant hot spots were mainly in the south and southwest parts of the province. For example, Wenzhou, which contains six counties, three districts, and two county-level cities, has been one of the strongest hot spots of TB since 2010. Also, Quzhou and Lishui were significant hot spots with the highest rates of TB notification during the study period (Figs. 2 & 3). On the contrary, Huzhou and Jiaxing were two significant cold spots located in the north and northeast parts of the province (Fig. 3).

### Space-time Clusters of TB cases

**Fig. 4:**
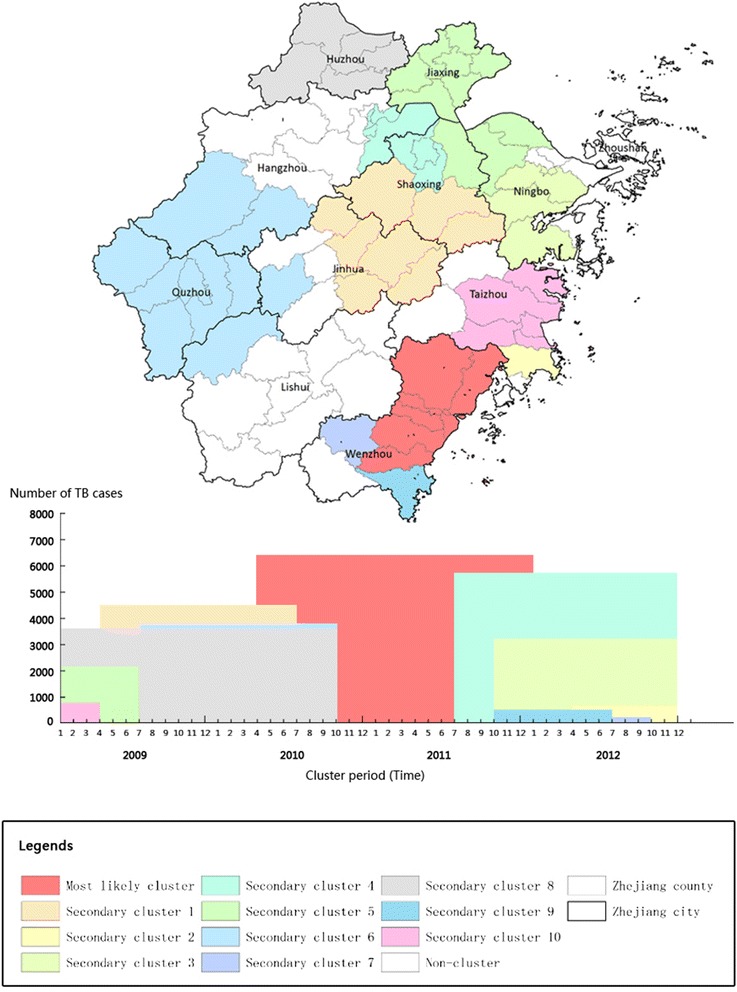
The space-time clusters of TB cases at the county level in Zhejiang province, 2009–2011

Table 3 showed the analysis results of space-time scan statistic, including one most likely cluster and ten secondary clusters. The main cluster was statistically significant with *p* < 0.001 and the ten clusters were *p* < 0.05. These space-time clusters were distinguished in different colour as shown in Fig. 4. The main cluster occupied large areas of the Wenzhou prefecture, which covered 7 counties and 6,375 TB cases. Such a significant cluster was long-term persistent from early spring 2010 to the end of 2011. Our result found that people within this cluster had 1.6 times higher risk of TB infection than those outside the hotspot (Relative risk: RR = 1.6, Table 3). In addition, we also found that around two-third secondary clusters occurred in winter and early spring and persisted around six months. The other one-third secondary clusters including counties in Shaoxing and Quzhou (secondary cluster 4 and 6, Table 3) located in north and west of Zhejiang, emerged in summer but lasted a longer period for more than twelve months. The relative risk ratio of these secondary clusters varied from 1.27 to 1.56.

**Table 3:** Space-time clusters of TB case in Zhejiang, China from 2009/1/1 to 2012/12/31 (setting 50 % of the population as the maximum spatial cluster size)

Cluster	Cluster period	Cluster center/Radius	Number of counties in the Cluster	Number of TB cases	RR
Most likely cluster	2010/4/1 to 2011/12/31	(27.903440 N, 121.113042 E)/55.76 km	7	6375	1.67
Secondary cluster 1	2009/4/1 to 2010/6/30	(29.233899 N, 120.369091 E)/53.89 km	8	4515	1.34
Secondary cluster 2	2012/4/1 to 2012/12/31	(28.398269 N, 121.407348 E)/0 km	1	795	1.43
Secondary cluster 3	2011/10/1 to 2012/12/31	(29.613659 N, 121.364938 E)/40.49 km	5	3067	1.31
Secondary cluster 4	2011/7/1 to 2012/12/31	(30.171950 N, 120.383971 E)/29.73 km	7	5830	1.36
Secondary cluster 5	2009/1/1 to 2009/6/30	(30.523916 N, 120.871586 E)/58.71 km	11	2114	1.39
Secondary cluster 6	2009/7/1 to 2010/3/31	(29.186764 N, 118.319280E)/117.02 km	10	3873	1.48
Secondary cluster 7	2012/7/1 to 2012/9/30	(27.809978 N, 120.016669 E)/0 km	1	96	1.56
Secondary cluster 8	2009/1/1 to 2010/9/30	(30.978934 N, 119.808732 E)/53.02 km	5	3711	1.28
Secondary cluster 9	2011/10/1 to 2012/6/30	(27.426300 N, 120.433287 E)/0 km	1	486	1.27
Secondary cluster 10	2009/1/1 to 2009/3/31	(28.855321 N, 121.204335 E)/39.52 km	5	688	1.31

Most likely cluster *P* value <0.001; Secondary cluster *P* values <0.05

*RR* relative risk

## Discussion

We studied the temporal trends of TB cases from 2009 to 2012 and examined the spatial patterns and space-time clusters at the county level in Zhejiang province using time series and advanced spatial data analyses. Through the time series study, seasonal trends were detected with apparent peaks in both summer and late autumn. In winter, the reduction of exposures to ultra violet from sunlight and the poor ventilation in indoor settings may increase the opportunity of infections with TB bacteria [25], thus most TB infections may occur in winter. However, the disease normally need time to develop symptoms in patients. In addition, patients may lack the knowledge of where to seek TB care in the health systems, while the referral from the general hospitals to TB designated clinics also delay TB diagnosis and treatment [26]. All these factors may contribute to the delay of TB diagnosis and reporting, and consequently, more TB infections were reported in summer than other seasons. Such seasonal patterns identified were consistent with previous studies in many regions such as other provinces in China [27], Hong Kong [28], Taiwan [29], India [25], and United Kingdom [30].

We applied Moran’s *I* and Local *G*_i_^*^ statistics to identify the global and local patterns of TB cases under the county level. The estimate of the Moran’s *I* was statistically significant ranging from 0.296 to 0.323 for all the four years, which indicated that TB infections were clustering geographically at the county level with similar notification rates. The statistically significant clusters, including hot spots and cold spots, were identified in the different parts of the province using Local *G*_i_^*^ statistic. One most likely cluster and ten secondary clusters were identified regarding the variations of the TB cases in space and time through using the space-time scan statistic. This method has been used widely to detect space-time clusters in many diseases, including hand-foot-mouth disease [31], malaria [32], dengue [33], and hemorrhagic fever [34]. These TB clusters identified were similar to that of the Local G_i_^*^ statistic. Both the results are highly consistent with a previous study [6].

It could be problematic if the high prevalence of TB infections were simply associated with low socio-economic status, although poverty was reported as one of factors for the high prevalence of TB notification in US [35, 36]. Wenzhou county is one of the wealthiest commercial regions in the province, but has a higher risk of TB infections than other counties in Zhejiang. The average TB notification rate of the province was 85.68 per 100,000 people [6], whereas the most likely cluster, including Longwan, Ouhai, and Yongjia counties, were much higher than the average, with 303.29, 300.60, and 138.22 per 100,000 people respectively, and the relative risk (RR) was 1.67 in the cluster of Wenzhou. This higher risk of TB infections may be related to migrants and limited healthcare and resources allocated for this specific population. For example, over 3.35 million migrant workers swarmed into Wenzhou in 2010, accounting for 41.5 % the total migrants of the province in that year [12]. During the period of 2010–2011, there were 2,480 migrant cases and 3,895 permanent residence cases in the most likely cluster of Wenzhou, whereas the notification rate of TB in migrants had reached 76.85 per 100,000 people in the cluster, a 1.5 times higher than that of permanent residence in the same area. Migrant workers are usually not entitled to social welfares and health insurance as local residents, which made them difficult to access healthcare service [37, 38]. According to our surveillance data, more than 50 % of the TB cases were young adults below 45 years and over one third of these cases were migrants. The actual rate of TB notification in migrants, however, might be underestimated, because migrants usually do not seek medical cares or seeking care in unqualified clinics due to the lack of health insurance and other protections [39, 40]. In addition, well-connected traffic networks and infrastructures could also be a factor in association with people’s migration and thus facilitating the transmission of TB in distance [15]. From this study, we may notice that solid socio-economic status could be associated with the reduction of TB notification rates, but a thorough healthcare and prevention system is crucial to be established for the control of the disease.

Those identified clusters descripted the process of TB dynamics in space and time. Initially, the secondary likely TB clusters were found in the areas of Huzhou, Taizhou, and Ningbo, the north coast province in between 2009 and early 2010. TB infections were found to keep transmitting from the north coast to the west and southeast inner areas and aggregated in Wenzhou and Quzhou counties during the period of 2010–2012. In early 2012, a few secondary likely clusters returned to the north and coastal areas such as Shaoxing and Taizhou counties. Among these clusters, Huzhou, a county in the north Zhejiang, was the earliest secondary likely cluster dominant for more than 18 months since its occurrence in January 2009. This county has been regarded as one of important hotspots to spread diseases such as avian influenza H5N1 and H7N9 viruses in China. In this study, we also found that this county has a high risk of TB infections with RR, 1.28. Compared to other secondary likely clusters, the cluster of Quzhou contained ten neighboring counties covering a large number of population and areas. This region is relatively poor in Zhejiang and has the secondary highest risk of TB infection (RR = 1.48, Table 3) after the most likely cluster in Wenzhou. Unlike the clusters of Wenzhou, Shaoxing, and Taizhou, the high risk of Quzhou cluster may be associated with its low economic status and limited healthcare resources [41]. Migrants from Zhejiang and other neighboring provinces such as Fujian, Jiangxi, and Anhui might have also contributed the long-term persistence of TB cluster in Quzhou [42]. These identified clusters differed in their socio-economic status, demography structures, and natural environment in association with mechanisms that drive the spread of TB geographically. Understanding the interaction of TB transmission, peoples’ migration, and society, albeit highly complex and dynamic, is very crucial for the prevention of TB infections and spreads.

This study was subject to some limitations. First, the estimated risk of TB infection might be underestimated in some areas because cases may not be reported or recorded in the official system. Second, we applied space-time scan statistic to detect clusters in different space and periods of time. The method that relies on circular spatial scanning windows and space-time cylinders does not allow for irregular space [43]. It is possible to make the identified clusters statistically unstable or varying with the changes of bandwidths (i.e., scaling dependence). This analysis result could be used to provide strategies for the prevention of TB at the county level, which is unique and differed from those identified at a provincial or nationwide level. The clusters identified by the space-time scan statistic and the Local *G*_i_^*^ statistic clusters were similar, which may indicate the robust of our analysis results, although it was limited by the methods applied. Finally, the present study only analyzed the spatial and temporal patterns of TB cases and clusters. High prevalence of TB may be related to both individual and socio-economic factors such as tobacco smoking [44], poor living environment and crowded housing [45, 46], low economic status [47], exposure to ambient air pollution [48], and some meteorological factors [7]. Further studies can be conducted to uncover the roles of these factors in the spread of TB disease.

## Conclusion

This study identified temporal trends and spatial distribution of TB cases in Zhejiang province using time series, spatial data analyses, and GIS. Poverty, migration, and seasonal effects may play important roles in potential clusters, which is informative to TB control and prevention.

## Additional file

Additional file 1:
**Multilingual abstracts in the six official working languages of the United Nations.** (PDF 548 kb)
