# Supplementary material for: Spatial and temporal analysis of tuberculosis in Zhejiang Province, China, 2009-2012
Source: Infect Dis Poverty. 2016 Feb 23;5:11. doi: 10.1186/s40249-016-0104-2 (PMC4763446; doi:10.1186/s40249-016-0104-2)
Supplement: Additional file 1: — Multilingual abstracts in the six official working languages of the United Nations. (PDF 548 kb) [file 40249_2016_104_MOESM1_ESM.pdf]

Translation of the abstract into the six official working languages of the United Nations

تحليل مكاني وزمني لمرض السل في مقاطعة تشيجيانغ في الصين من سنة 2009 إلى سنة 2012

إرجيا جي وكسين زهانغ وكسياومنغ وانغ وكسياولين واي

ملخص

يعتبر السل مشكلة صحية عامة رئيسية. لقد أظهر المرض تغيرا كبيرا في المكان والزمان. تهدف هذه الدراسة إلى بحث الديناميكية الزمانية والمكانية لحالات مرضى السل في إطار ديناميكية إقتصادية وجغرافية لمقاطعة في الصين مع إشارات محددة لمراقبة السل لصناع السياسة. لقد تم تسجيل وتجميع المعطيات الخاصة بحالات مرض السل من سنة 2009 إلى سنة 2012 من برنامج مرض السل في مقاطعة تشيجيانغ لفائدة مركز مراقبة الأمراض المعدية والوقاية منها. لقد اعتمدنا طريقة تحليل معطيات التسلسل الزمني والاستكشاف المكاني بما فيها طريقة موران I الإحصائية وطريقة جتيس جي وكولدورف للمسح الزماني والمكاني من أجل تحديد التوجهات الزمنية الظرفية وما يرتبط بها من آثار على مستوى المكان في المحافظات. لقد تم تسجيل 147,941 حالة سل ما بين 2009 إلى سنة 2012 في مقاطعة تشيجيانغ. يعتبر الشباب من الذكور هم الأعلى نسبة في هذا الإحصاء وهم من المقيمين الدائمين. لقد تم تسجيل أكثر الحالات في شهر أبريل مع وجود ارتفاع محدود في شهر يونيو ويوليو وأكتوبر. لقد كان هذا المرض يتراوح–حسب معايير موران I- مابين 0،29 إلى 0،32 ($p < 0.001$). لقد تم التعرف على المجموعة التي انتشر فيها المرض و7 مجموعات أخرى فرعية متمركزة في الجنوب الشرقي والغربي للمقاطعة. لقد حددت هذه الدراسة الارتباطات الموسمية والتجمعات الزمنية والمكانية الهامة لمرضى السل في مقاطعة تشيجيانغ. قد يلعب الفقر والهجرة والعوامل الموسمية دورا هاما في بعض التجمعات.

Translated from English version into Arabic by malika2012, through

基于中国浙江省 2009-2012 年肺结核病的时空分析

葛尔佳，张鑫，王晓萌，魏晓林

摘要

肺结核是公共卫生领域主要所关注的传染病之一。该疾病在空间和时间上都具有很大的差异性。我们的研究主要是探测该病在一个经济发展与地理多样性地区的时空变化，从而为决策者提供结核病防治策略的依据。数据来源于浙江省疾病控制中心收集并提供的 2009-2012 年全省报告的共 147,941 例结核病病例。我们利用时间序列和空间分析，其中包括 Moran’s I, Local Getis’s G_i^*, and Kulldorff’s space-time scan statistics 等空间统计方法，在县级尺度上对结核病的时间趋势与空间分布模式进行了探测。研究发现浙江省结核病患者主要是年轻人（15-29 岁），男性，和常住居民。结核病报告多发于每年四月，并在六，七，和十月出现发病小高峰。同时，我们计算出 Moran’s I 的值在 0.29 - 0.32 范围内，表明结核病的发病具有显著的空间聚合性。该疾病的时空热点区域主要集中在浙江省的东南与西部县区。贫穷，外来民工，以及季节因素对结核病的爆发与传播具有重要作用，形成该疾病热点区域。

Translated from English version into Chinese by Ge Erjia, Wei Xiaolin

Analyse spatiale et temporelle de la tuberculose dans la province chinoise du Zhejiang, 2009-2012

Erjia Ge, Xin Zhang, Xiaomeng Wang, Xiaolin Wei

Résumé

La tuberculose est une maladie infectieuse et un problème majeur de santé publique. Elle a connu de grandes variations dans l'espace et le temps. La présente étude explore la dynamique spatiale et temporelle des cas de tuberculose dans une province de Chine économiquement dynamique et géographiquement bien située, en ciblant spécifiquement la lutte contre la maladie à l'intention des décideurs politiques. Les données de tous les cas déclarés de tuberculose rapportés entre 2009 et 2012 sont tirées du programme de lutte contre la tuberculose du Centre provincial de lutte contre les maladies et de prévention du Zhejiang. Nous avons utilisé des analyses de séries temporelles et de données spatiales exploratoires, en utilisant notamment l'indice *I* de Moran, l'indice G_i^* local de Geti et les statistiques de balayage spatiotemporel de Kulldorff pour identifier les tendances dans le temps et les schémas spatiaux de la tuberculose à l'échelle du comté. Au total, 147 941 cas de tuberculose ont été rapportés dans le Zhejiang entre 2009 et 2012. Une proportion élevée de ces patients étaient des hommes jeunes, inscrits comme résidents permanents dans la province. Les cas de tuberculoses étaient plus fréquemment rapportés en avril, avec d'autres petits pics en juin, juillet et octobre. Les cas se regroupaient dans l'espace, avec des valeurs de l'indice *I* de Moran comprises entre 0,29 et 0,32 (p < 0,001). Un regroupement très probable et dix regroupements secondaires ont été identifiés, principalement concentrés dans les comtés situés dans le sud-est et l'ouest de la province. L'étude a identifié des schémas saisonniers et des regroupements significatifs des cas de tuberculose dans le temps et l'espace dans la province chinoise du Zhejiang. Il se peut que la pauvreté, les migrations et des effets saisonniers jouent un rôle important dans les regroupements potentiels.

Translated from English version into French by Myriam Grandchamp, through

TRANSLATORS WITHOUT BORDERS

Пространственный и временной анализ туберкулеза в провинции Чжэцзян, Китай, 2009-2012 г.г.

Эрцзя Ге, Ксин Жанг, Ксяомен Ванг, Ксяолин Вей

РЕФЕРАТ

Туберкулез (ТВ) - это инфекционное заболевание, представляющее серьезную угрозу здоровью общества. Это заболевание продемонстрировало значительную пространственно-временную изменчивость. Целью данного исследования является изучение пространственно-временной динамики случаев заболевания туберкулезом в экономически и географически динамичной провинции Китая с разработкой рекомендаций по борьбе с туберкулезом для директивных органов. Были собраны данные по всем зарегистрированным случаям туберкулеза с 2009 по 2012 год в рамках программы по борьбе с туберкулезом Санитарно-эпидемиологического центра провинции Чжэцзян. Нами использовались анализ временных рядов и оценочный анализ пространственных характеристик, включая локальный индекс Морана *I*, локальную статистику ГетисаG_i^* и and пространственно-временную статистику Кулдорфа для определения тенденции во времени и пространственных диаграмм туберкулеза на уровне страны. Всего в Чжэцзяне за

2009-2012 г.г. зарегистрировано 147941 случаев туберкулеза. Большую часть случаев заболевания туберкулезом представляли молодые мужчины, постоянно проживающие в данной провинции. Случаи заболевания туберкулезом чаще всего отмечались в апреле с небольшими вспышками в июне, июле и октябре. Для данного заболевания отмечена пространственная кластеризация со значениями локального индекса Морана *I* в диапазоне от 0,29 до 0,32 (p < 0,001). Были выделены наиболее вероятный кластер и десять второстепенных кластеров, в основном сконцентрированные в юго-восточных и западных областях провинции. Данное исследование определило характер сезонных изменений и значимые пространственно-временные кластеры случаев заболевания туберкулезом в провинции Чжэцзян в Китае. Бедность, миграции и действие сезонных факторов играют важную роль в потенциальных кластерах.

Translated from English version into Russian by Alena Hrybouskaya, through

TRANSLATORS WITHOUT BORDERS

Análisis espacial y temporal de la tuberculosis en la provincia de Zhejiang, China, 2009-2012

Erjia Ge, Xin Zhang, Xiaomeng Wang, Xiaolin Wei

RESUMEN

La tuberculosis (TB) es una enfermedad infecciosa de gran interés en el ámbito de la salud pública. La enfermedad ha demostrado grandes variaciones en tiempo y espacio. Este estudio tiene como objetivo explorar la dinámica temporo-espacial de los casos de tuberculosis en una provincia de China económica y geográficamente dinámica con referencias específicas al control de la TB para quienes determinan las políticas a seguir. La información sobre todos los casos reportados de TB desde el año 2009 al año 2012 se recolectó del programa de TB del Centro Provincial de Control y Prevención de Enfermedades de Zhejiang. Utilizamos análisis exploratorios de datos espaciales y basados en series de tiempo, incluyendo las estadísticas temporo-espaciales I de Moran, la G de Getis y de Kulldorff, para identificar las tendencias temporales y los patrones espaciales de TB a nivel de condado. Durante los años 2009 a 2012 se reportaron en Zhejiang un total de 147.941 casos de TB. De todos los casos de TB notificados en la provincia, una proporción alta de los casos de TB fueron hombres jóvenes, y residentes permanentes registrados. Los casos de TB fueron más frecuentemente reportados en abril con pequeños picos en junio, julio y octubre. Esta enfermedad, con la técnica de Moran, mostraba conglomerados espaciales con valores que variaban entre 0,29 y 0,32 (p < 0,001). Se identificó un conglomerado más frecuente y diez conglomerados secundarios, concentrados principalmente en los condados al sureste y oeste de la provincia. Este estudio identificó patrones estacionales y conglomerados temporo-espaciales significativos de los casos de TB en Zhejiang, China. Es posible que la pobreza, la inmigración y los efectos estacionales jueguen un rol importante en los posibles conglomerados.

Translated from English version into Spanish by Maria Alejandra Aguada, through

TRANSLATORS WITHOUT BORDERS
